# Wafer‐Scale 3D Integration for High‐Density Multi‐Valued Neuromorphic Logic

**DOI:** 10.1002/advs.77437

**Published:** 2026-08-25

**Authors:** Chang‐Hyeon Kim, Min Seok Kim, Joong Bum Rhim, Tae In Kim, Hyuck‐In Kwon, Ick‐Joon Park

**Affiliations:** ^1^ School of Electrical and Electronics Engineering Chung‐Ang University Seoul South Korea; ^2^ Department of Electronics and Information Engineering Hansung University Seoul South Korea; ^3^ Department of Electrical Engineering and Computer Science Massachusetts Institute of Technology Cambridge Massachusetts USA; ^4^ School of Electrical and Electronic Engineering Program in Semiconductor Convergence Inha University Incheon South Korea

**Keywords:** 3D monolithic integration, heterojunction FET, high‐density ternary circuit, multi‐valued logic, neuromorphic logic, tellurium transistor

## Abstract

As conventional transistor scaling approaches fundamental limits, multi‐valued logic (MVL) has emerged as a promising strategy to enhance information density and reduce circuit complexity beyond binary complementary metal‐oxide‐semiconductor (CMOS) technology. Here, we present a monolithic three‐dimensional (3D) vertically stacked MVL architecture based on a tellurium (Te)/indium‐gallium‐zinc‐oxide (IGZO) heterojunction field‐effect transistor (H‐FET) integrated with a crystallinity‐enhanced Te field‐effect transistor (CE‐Te FET). Engineered interfacial band alignment in the H‐FET induces carrier confinement and gate‐tunable electron‐dominated transport, enabling intrinsic ternary switching through controlled current modulation. Complementary channel engineering of the CE‐Te FET via oxidation and crystallinity recovery suppresses off‐state leakage and optimizes transconductance matching, stabilizing the intermediate logic state. Using low‐temperature CMOS‐compatible processes, we realize wafer‐scale vertically stacked ternary circuits exhibiting robust three‐level operation, high uniformity, and long‐term stability. System‐level analysis reveals substantial gains in logic density and area efficiency enabled by H‐FET‐assisted 3D integration. Furthermore, the ternary voltage characteristics are directly mapped to ternary weight neural networks, enabling high‐accuracy handwritten digit classification. This scalable inorganic 3D MVL platform establishes a practical pathway toward high‐density logic and multi‐valued neuromorphic computing architectures beyond conventional binary planar scaling.

## Graphical abstract

Wafer‐scale 3D vertically integrated multi‐valued logic (MVL) circuits are realized by monolithically integrating a Te/IGZO heterojunction FET with an engineered Te pull‐up FET. Heterointerface‐mediated transport and optimized current matching enable stable ternary operation, while vertical stacking reduces circuit footprint and interconnect complexity, providing a scalable platform for high‐density multi‐valued information processing and ternary‐weight neuromorphic computing.

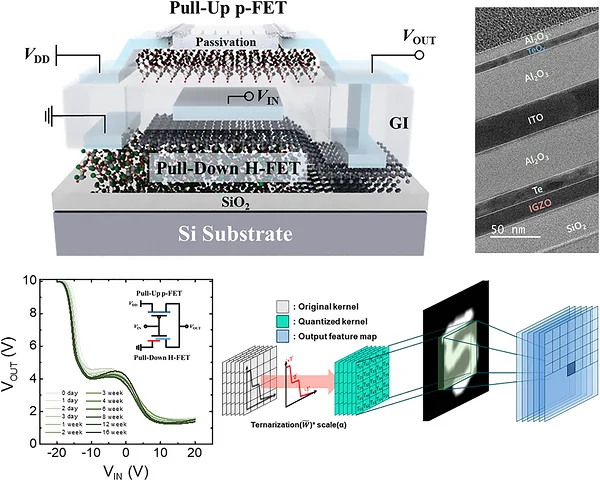

## 1 Introduction

Continued scaling of transistor dimensions has driven remarkable improvements in computing performance and integration density over past decades. However, as device geometries approach fundamental physical limits, further gains through conventional miniaturization are becoming increasingly constrained [1, 2, 3]. This has prompted intensive exploration of alternative device architectures and computing paradigms capable of delivering higher information density and energy efficiency beyond binary logic systems [4, 5, 6]. Multi‐valued logic (MVL), which extends digital representation beyond two discrete states, has emerged as a promising strategy to enhance computational density while reducing circuit complexity and interconnect overhead [6, 7, 8]. In particular, ternary logic systems theoretically enable a substantial reduction in device count and wiring requirements compared to binary architectures, offering improved scalability for advanced integrated circuits [7, 8, 9]. In contrast to binary gates, MVL gates introduce additional intermediate states, enabling higher information density with reduced device interconnectivity. For example, a ternary logic system can reduce system complexity to approximately 63% while maintaining the same information capacity [7]. Moreover, the three distinct logic levels of ternary circuits naturally align with ternary weight representations in neural networks, providing an attractive hardware platform for neuromorphic computing and data‐intensive applications [10, 11, 12].

Realizing practical MVL circuits, however, requires device‐level mechanisms capable of generating stable intermediate states within a compact footprint. Conventional complementary metal‐oxide‐semiconductor (CMOS) implementations of ternary logic typically require multiple additional transistors, inevitably increasing circuit complexity and power consumption [13, 14]. To address these limitations, heterojunction‐based transistors exhibiting negative differential resistance or negative transconductance have been explored using diverse material platforms, including two‐dimensional materials and organic semiconductors [15, 16, 17, 18, 19]. Since heterojunction‐based transistors exploit non‐monotonic transfer characteristics, this can enable intrinsic multi‐state operation and realize ternary logic functionality within a single device. By engineering carrier transport across p‐n heterointerfaces, such devices can exhibit gate‐controlled current modulation regimes that support intermediate output levels without the need for additional transistors, thereby simplifying circuit design and reducing power consumption [20, 21, 22, 23, 24, 25, 26, 27, 28].

Furthermore, beyond device‐level innovation, three‐dimensional (3D) vertical integration offers an additional pathway to dramatically increase logic density without shrinking lateral device dimensions. Vertically stacked transistor architectures have been successfully demonstrated in organic and oxide electronic systems, enabling higher transistor counts per unit area while preserving device performance and uniformity [29, 30, 31, 32]. In the context of MVL, combining intrinsic multi‐state devices with vertical stacking architectures presents a powerful approach to further amplify information density, reduce interconnect complexity, and realize compact multifunctional logic circuits [11, 23]. Recent studies have demonstrated vertically stacked ternary inverters based on organic heterojunction devices and floating‐gate‐assisted architectures, achieving stable intermediate states and improved integration density [23]. Parallel efforts have explored vertically integrated ternary circuits using electrochemical and organic transistor platforms, extending MVL concepts toward neuromorphic hardware implementations [11]. While these works underscore the promise of combining MVL with 3D integration, they still suffer from limited electrical stability, scalability challenges, or incompatibility with CMOS‐compatible fabrication processes [33, 34].

Among various material platforms, oxide semiconductor and van‐der‐Waals semiconductor heterojunctions offer distinct advantages including low‐temperature processability, excellent electrical stability, high uniformity, and compatibility with large‐area fabrication techniques, positioning them as a robust foundation for scalable MVL implementation [26, 35, 36]. In particular, group VI tellurium (Te)‐based semiconductors provide high intrinsic carrier mobility and excellent p‐type conduction, enabling efficient heterointerface transport engineering for multi‐valued operation [37, 38, 39, 40]. Integrating such oxide‐ and Te‐based transistors in vertically stacked architectures offers a compelling pathway toward high‐density ternary logic with enhanced scalability and CMOS compatibility. However, existing implementations have largely relied on planar device configurations, which limit achievable logic density and system‐level scalability, and demonstrations of 3D vertical ternary logic circuits have so far remained largely unexplored [26, 27, 28]. Accordingly, vertically integrated inorganic ternary logic architectures that combine p‐type Te with n‐type indium‐gallium‐zinc‐oxide (IGZO) offer a promising route to reliable non‐monotonic transport and stable ternary inverter operation via interfacial transport engineering. Despite substantial progress in heterojunction NDT devices and ternary inverters based on two‐dimensional and organic semiconductors, most previous studies have focused on either intrinsic multi‐state transport or planar circuit implementation (Table S1) [17, 20, 21, 22, 23, 24, 25, 26, 27, 28, 29, 30]. The translation of intrinsic MVL functionality into a monolithically integrated vertical logic architecture with a quantified system‐level density advantage remains insufficiently explored. Here, we address this gap by translating intrinsic heterojunction‐enabled MVL operation into a monolithically integrated vertical logic architecture through the vertical integration of a Te/IGZO H‐FET and a CE‐Te FET using a low‐temperature, wafer‐scale fabrication process.

In this Article, we report a monolithic 3D vertically stacked MVL architecture that integrates a Te/IGZO heterojunction field‐effect transistor (H‐FET) as the pull‐down device with a crystallinity‐enhanced Te field‐effect transistor (CE‐Te FET) as the pull‐up transistor in a compact vertical configuration. Interfacial band alignment engineering induces carrier confinement and gate‐tunable electron‐dominated transport in the H‐FET, enabling intrinsic ternary switching behavior through controlled current modulation. Complementary channel engineering of the pull‐up transistor via UV‐ozone‐induced oxidation followed by Al_2_O_3_‐mediated crystallinity recovery effectively suppresses off‐state leakage and optimizes transconductance matching, which is critical for stabilizing the intermediate logic state. Using entirely low‐temperature CMOS‐compatible processes, we realize wafer‐scale vertically stacked ternary logics exhibiting robust three‐level operation, high device‐to‐device uniformity, and long‐term environmental stability. Beyond logic demonstration, we further integrate the ternary voltage characteristics into ternary weight neural (TWN) networks for handwritten digit recognition, illustrating the applicability of the proposed MVL architecture to neuromorphic computing workloads. System‐level analysis reveals substantial gains in logic density and area efficiency enabled by the combination of intrinsic multi‐valued operation and 3D vertical integration. Together, these results establish a scalable inorganic semiconductor platform for vertically integrated MVL, providing a practical pathway toward high‐density computing architectures that transcend the limitations of conventional binary CMOS technology.

## 2 Results and Discussion

### 2.1 Monolithic Vertically Stacked MVL Architecture

The schematic illustration of the 3D vertically stacked MVL device is presented in Figure 1a. The pull‐down Te/IGZO H‐FET and pull‐up p‐type CE‐Te FET are monolithically integrated along the out‐of‐plane direction. By confining complementary pull‐up and pull‐down functions to the vertical dimension, this architecture converts heterojunction‐level band engineering into circuit‐level MVL through vertical current competition, enabling high‐density 3D integration and energy‐efficient multi‐valued computing beyond planar scaling limits. Figure 1b shows the circuit representation of the proposed structure. The pull‐up CE‐Te FET connects the output node (*V*_OUT_) to the supply voltage (*V*_DD_), while the pull‐down Te/IGZO H‐FET connects *V*_OUT_ to ground. Under a common input bias, the circuit stabilizes at three distinct output states including high, intermediate, and low states, arising from the controlled balance between the pull‐up and pull‐down currents. Figure 1c presents the fabrication flow of the vertically integrated MVL device, visualized by a series of OM images corresponding to ten major process steps, all performed at temperatures not exceeding 200°C. This sequence enables monolithic vertical integration while maintaining full compatibility with low‐temperature back‐end‐of‐line processing. The OM image in Figure S1 further illustrates the overall dimensions and footprint of the fully fabricated MVL device. Figure 1d presents a cross‐sectional field‐emission transmission electron microscopy (FE‐TEM) image of the fully integrated vertical stack. All functional layers are clearly resolved, exhibiting void‐free interfaces and conformal film coverage across the entire stack, confirming the structural integrity of the monolithic 3D architecture. The energy‐dispersive X‐ray spectroscopy (EDS) elemental mappings in Figure 1e also verify the well‐defined vertical distribution of Te, In, Ga, Zn, O, Al, and Sn, corroborating the intended layer sequence and compositional uniformity without detectable interlayer mixing. Figure 1f–h show 3D atomic force microscopy (AFM) images of the pull‐down IGZO, pull‐down Te, and oxidized pull‐up Te layers, respectively. All channel layers exhibit continuous surfaces with well‐defined topographies, indicating uniform film growth suitable for reliable vertical current modulation. The corresponding root‐mean‐square (RMS) surface roughness values are 0.681 nm, 1.54 nm, and 0.818 nm for the pull‐down IGZO, pull‐down Te, and pull‐up Te layers, respectively, confirming the smooth and uniform surfaces of the deposited films. In addition, the corresponding height profiles of the AFM images are provided in Figure S2a–c, confirming thicknesses of approximately 20 nm for the pull‐down IGZO, 10 nm for the pull‐down Te, and 8 nm for the pull‐up Te layers, respectively. Together, these structural and morphological analyses confirm that the proposed low‐temperature fabrication process yields a high‐quality, vertically stacked heterostructure, providing a solid physical foundation for the MVL operation demonstrated in the subsequent sections. Importantly, the proposed architecture enhances system‐level logic density through two complementary mechanisms: (i) intrinsic ternary operation, which reduces the required transistor count, and (ii) monolithic vertical integration, which compresses the lateral footprint. This combination distinguishes the proposed platform from previously reported heterojunction NDT devices and representative 2D‐ and organic‐semiconductor MVL implementations, providing the architectural foundation for high‐density logic integration.

**FIGURE 1:**
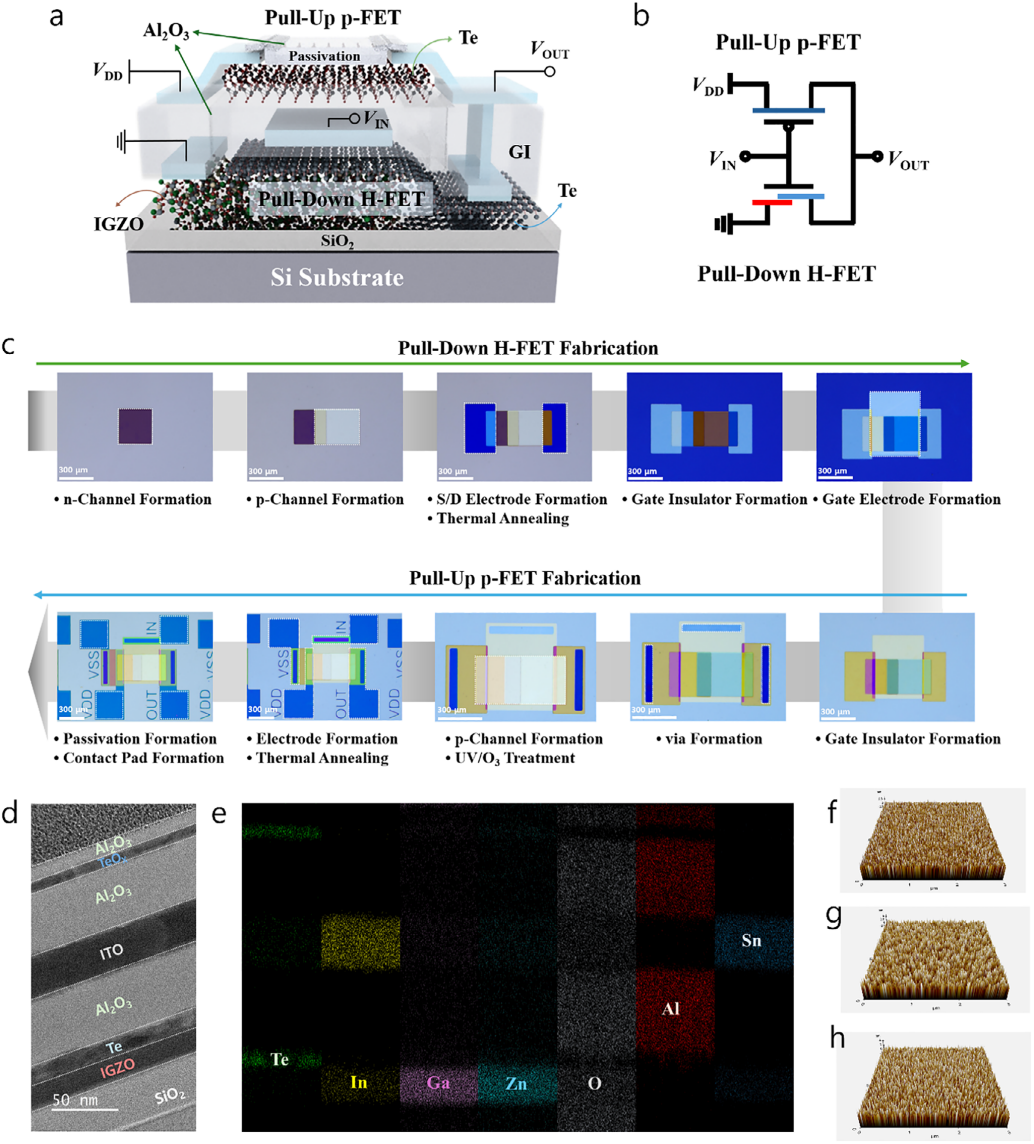
Monolithic 3D vertically stacked multi‐valued logic device architecture, fabrication, and structural characterization. (a) Schematic illustration of the 3D vertically stacked MVL device, consisting of a bottom pull‐down H‐FET based on a Te/IGZO heterointerface and a top pull‐up CE‐Te FET, monolithically integrated along the vertical direction. (b) Circuit representation of the vertically integrated MVL unit. (c) Optical microscopy (OM) images showing the fabrication process flow of the vertically stacked device, comprising ten major steps performed entirely at temperatures below 200 °C. (d) Cross‐sectional TEM image of the fully integrated vertical stack and (e) its corresponding EDS elemental mapping. The scale bar represents 50 nm. (f–h) 3D AFM images of the pull‐down IGZO layer (f), pull‐down Te layer (g), and pull‐up Te layer (h).

### 2.2 Interfacial Band Alignment and Transport in the Te/IGZO H‐FET

Figure 2a shows a schematic illustration of the pull‐down H‐FET based on the Te/IGZO heterostructure. The device utilizes the partial overlap between the p‐type Te layer and the n‐type IGZO layer to enable gate‐modulated charge transport across the heterointerface, forming the basis of the pull‐down operation in the vertically stacked logic architecture. Figure 2b presents the Raman spectra of pristine Te, IGZO, and the Te/IGZO heterojunction. The Te film exhibits the characteristic A_1_ and E_2_ vibrational modes observed at 122.66 and 141.66 cm^−1^, respectively, confirming the preservation of the quasi‐two‐dimensional helical‐chain crystal structure of tellurium. In crystalline Te, the A_1_ and E_2_ modes originate from the expansion of trigonal helical chains and their asymmetric c‐axis stretching vibrations, respectively, serving as robust spectroscopic signatures of the quasi‐two‐dimensional Te lattice [41]. In the Te/IGZO heterojunction, both A_1_ and E_2_ modes show a slight blueshift compared to pristine Te, while maintaining nearly identical spectral features and peak separation, indicating that the Te lattice remains largely intact upon heterojunction formation. The slight blueshifts observed in the A_1_ and E_2_ peaks at the Te/IGZO heterojunction can be attributed to interfacial electronic coupling and local bonding modulation at the Te/IGZO interface, rather than to significant lattice distortion or strain, as evidenced by the preserved peak positions and spectral profiles compared with pristine Te (Figure S3) [42]. The crystalline characteristics are further examined by X‐ray diffraction (XRD) analysis in Figure 2c. The IGZO film exhibits a fully amorphous nature, whereas both Te and Te/IGZO samples display distinct diffraction peaks of hexagonal Te. The diffraction peaks are observed at 2θ = 23.04°, 27.62°, 40.55°, and 49.8°, which are indexed to the (100), (101), (110), and (021) crystallographic planes, respectively, in good agreement with previously reported quasi‐two‐dimensional Te thin films prepared by sputtering (diffraction peak card#04‐027‐7719). The full width at half maximum (FWHM) of the dominant Te diffraction peak increases slightly from 1.24 for pristine Te to 1.38 for Te/IGZO, suggesting a modest reduction in crystalline coherence induced by heterointerface formation. Using the Debye‐Scherrer relation, the average grain sizes are estimated to be 6.82 nm for Te and 6.12 nm for Te/IGZO, respectively. These results indicate that the integration of IGZO does not disrupt the fundamental crystalline phase of Te, while introducing subtle structural modulation consistent with heterojunction coupling.

**FIGURE 2:**
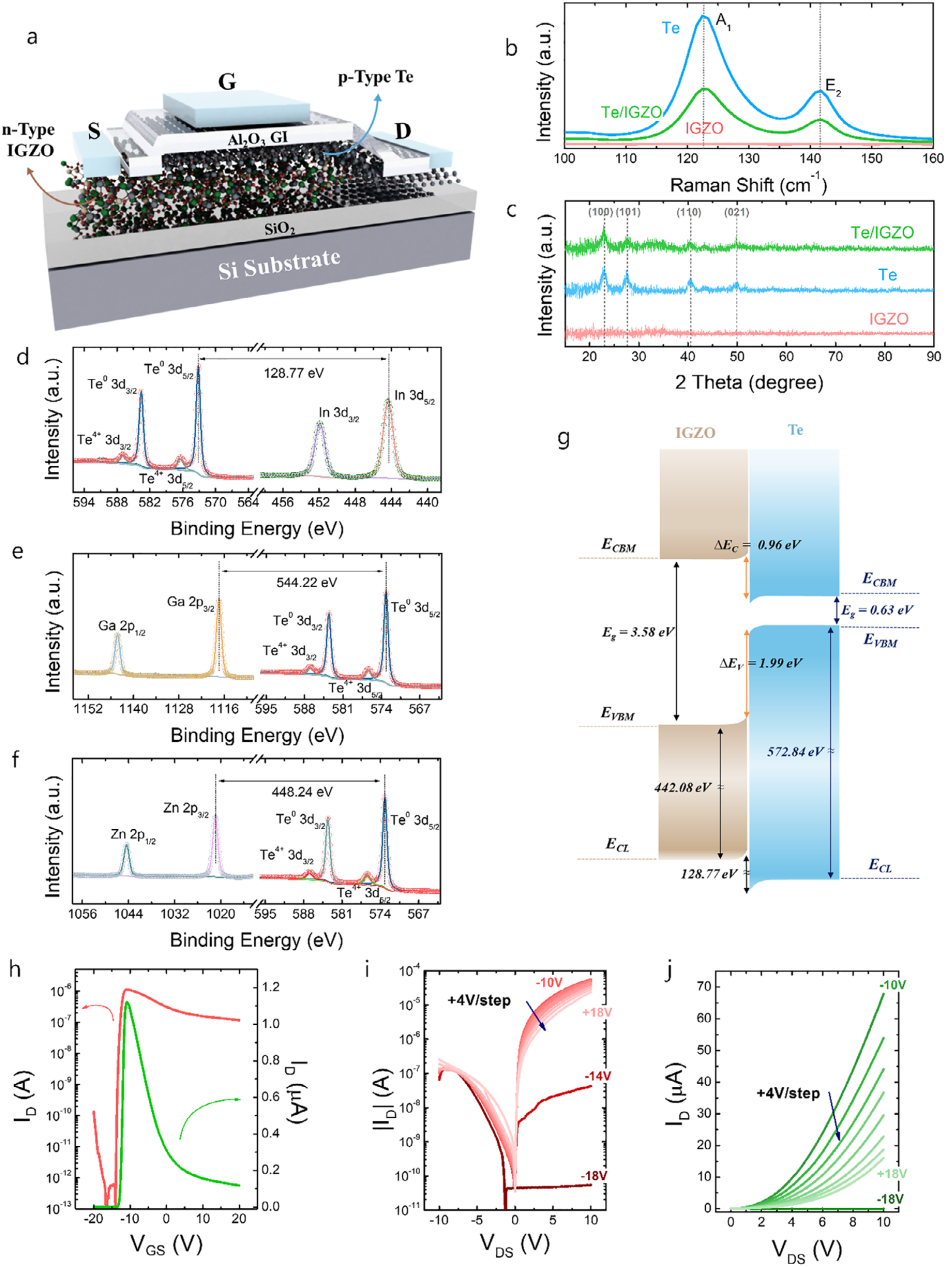
Structural, spectroscopic, band alignment, and electrical characteristics of the Te/IGZO H‐FET. (a) Schematic illustration of the Te/IGZO H‐FET, consisting of a p‐type Te layer vertically coupled with an n‐type IGZO layer to form a gate‐modulated pull‐down device. (b) Raman spectra of pristine Te, IGZO, and the Te/IGZO heterojunction, showing the characteristic A_1_ and E_2_ vibrational modes of crystalline Te. (c) XRD patterns of Te, IGZO, and Te/IGZO heterojunction films, where IGZO exhibits an amorphous structure while Te and Te/IGZO display distinct hexagonal Te diffraction peaks. (d–f) XPS core‐level spectra of the Te/IGZO heterojunction used for band alignment analysis: Te 3d and In 3d (d), Ga 2p and Te 3d (e), and Zn 2p and Te 3d (f). (g) Schematic band alignment diagram of the Te/IGZO heterointerface, revealing a type‐I straddling alignment. (h), Transfer characteristics of the Te/IGZO H‐FET, exhibiting gate‐modulated electron‐dominated conduction and non‐monotonic behavior arising from interfacial transport mechanisms. (i,j) Output characteristics of the Te/IGZO H‐FET measured at different gate voltages, presented in logarithmic (i) and linear (j) scales.

Figure 2d–f present a high‐resolution X‐ray photoelectron spectroscopy (XPS) analysis of the Te/IGZO heterojunction to quantitatively determine the interfacial band alignment [43, 44]. Figure 2d presents the core‐level XPS spectra of Te 3d and In 3d acquired from the Te/IGZO heterostructure, where the Te 3d_5/2_ are located at 573.15 eV, whereas the In 3d_5/2_ peak appears at 444.38 eV. Figure 2e,f display the corresponding Ga 2p–Te 3d and Zn 2p–Te 3d spectra, respectively, with the Ga 2p_3/2_ and Zn 2p_3/2_ peaks positioned at 1117.37 and 1021.39 eV, together with the Te 3d_5/2_ reference peak. The simultaneous observation of Te and IGZO‐related core levels confirms the formation of a chemically well‐defined heterointerface suitable for quantitative band offset extraction. To determine the band offsets at the heterojunction, the binding energy separations between the Te 3d peak and the valence band maximum (VBM) of bulk Te, as well as those between the IGZO core levels and the VBM of bulk IGZO, were independently obtained from reference samples (Figures S4 and S5). The valence band offset (VBO) was extracted following equation, expressed as

$$\Delta E_{V}=\left(E_{\mathrm{CL}}^{\mathrm{Te}}-E_{V}^{\mathrm{Te}}\right)-\left(E_{\mathrm{CL}}^{\mathrm{IGZO}}-E_{V}^{\mathrm{IGZO}}\right)-{\left(E_{\mathrm{CL}}^{\mathrm{Te}}-E_{\mathrm{CL}}^{\mathrm{IGZO}}\right)}_{\mathrm{interface}},$$

where *E*_CL_ exhibits the binding energy of the selected core level and *E_V_* represents the VBM. From the bulk Te sample, $E_{\mathrm{CL}}^{\mathrm{Te}}-E_{V}^{\mathrm{Te}}$ of 572.84 eV was obtained, while the $E_{\mathrm{CL}}^{\mathrm{IGZO}}-E_{V}^{\mathrm{IGZO}}$ of 442.08 eV (In 3d), 1115.06 eV (Ga 2p), and 1019.07 eV (Zn 2p) were extracted from the bulk IGZO XPS spectra. Multiple IGZO core levels (In, Ga, and Zn) were independently analyzed to ensure the robustness of the extracted band offsets against element‐specific chemical shifts and local coordination variations inherent to multicomponent amorphous oxides. The consistency among the offsets derived from different core‐level pairs confirms the reliability of the band alignment analysis and minimizes uncertainty arising from compositional or bonding inhomogeneity within the IGZO layer. The conduction band offset (CBO) was subsequently determined using the relation

$\Delta E_{C}=E_{g}^{\mathrm{IGZO}}\;-E_{g}^{\mathrm{Te}}-\Delta E_{V}$ where the bandgap energies of Te (0.63 eV) and IGZO (3.58 eV) were independently extracted from UV–vis absorption‐based Tauc plots (Figure S6). Based on this analysis, the Te/IGZO heterointerface exhibits a VBO value of 1.99 ± 0.05 eV and a CBO value of 0.96 ± 0.05 eV, establishing a type‐I straddling band alignment, as schematically illustrated in Figure 2g. The experimentally extracted band offsets inherently include the effects of interfacial band bending and dipole formation at the Te/IGZO heterointerface, which further modulate the local electrostatic potential without altering the type‐I alignment. This band alignment enables gate‐controllable current competition rather than binary switching, thereby establishing a stable intermediate operating regime. The large VBO renders hole injection across the Te/IGZO heterointerface energetically unfavorable, strongly suppressing hole transfer from the Te layer into IGZO. Furthermore, the valence band of IGZO exhibits pronounced localization, leading to a large effective hole mass and intrinsically poor hole transport even under conditions of partial hole accumulation [26, 27, 42]. As a result, hole‐mediated conduction is effectively inhibited within the heterojunction. In contrast, the conduction band profile permits electron transport to be selectively activated and continuously tuned by the gate electric field, making electrons the dominant carriers governing interfacial conduction. The type‐I band alignment further induces carrier confinement near the heterointerface, which localizes the transport pathway, suppresses bulk leakage channels, and enhances the sensitivity of current modulation. This confinement is critical for establishing a stable current balance that defines the intermediate logic state in MVL operation. The resulting asymmetry in carrier transport enforces an electron‐dominated pull‐down conduction mechanism, stabilizing the pull‐down operation and defining the fundamental charge‐transport behavior of the Te/IGZO heterojunction device. Based on this carrier‐selective type‐I band alignment, the continuously tunable electron current can be precisely balanced against the vertically integrated pull‐up CE‐Te FET, thereby naturally stabilizing a third logic state and forming the physical foundation for robust MVL functionality.

Figure 2h shows the representative transfer characteristics of the Te/IGZO H‐FET obtained at a drain‐to‐source voltage (*V*_DS_) of 1 V, which exhibits a notable negative differential transconductance (NDT) behavior. The charge transport in the Te/IGZO H‐FET emerges from the gate‐controlled interplay between an n‐type IGZO channel, a p‐type Te channel, and the rectifying Te/IGZO p‐n heterointerface formed between them. In contrast to the non‐monotonic response of the H‐FET, the individual Te and IGZO FETs exhibit conventional monotonic p‐type and n‐type transfer characteristics, respectively, without NDT behavior (Figure S7). Rather than operating as a conventional single‐channel transistor, the device exhibits a composite transport behavior governed by three different mechanisms: (i) At low gate‐to‐source voltage (*V*_GS_) below the threshold voltage of the IGZO FET (*V*_TH_n_), the electrostatic potential in the IGZO layer prevents electron accumulation, resulting in a highly resistive state as being fully depleted where carrier injection toward the heterointerface is effectively blocked. Under this condition, the heterojunction remains inactive and the drain current (*I*_D_) is strongly suppressed due to the absence of mobile electrons. (ii) As the *V*_GS_ exceeds *V*_TH_n_, electrons accumulate in the IGZO layer and are driven toward the heterointerface under an applied drain bias. This forward‐biased condition activates interfacial charge transfer, allowing electrons to diffuse into the Te region where they contribute to current flow through recombination‐mediated transport. Consequently, a rapid increase in the *I*_D_ can be achieved in this regime. (iii) Upon further gate biasing, where *V*_GS_ is beyond the threshold voltage of the Te FET (*V*_TH_p_), strong electrostatic depletion in the Te layer progressively reduces the hole population near the heterointerface, characterized by the emergence of negative differential transconductance behavior. Because interfacial transport relies on the presence of holes to sustain recombination‐driven electron flow, the diminishing hole density increasingly restricts carrier transfer across the junction. At the same time, the conductivity of the Te channel decreases as it approaches the off state. These effects collectively result in a decline in *I*_D_ despite increasing gate voltage, giving rise to a non‐monotonic transfer response. This sequential evolution defines the unique operational behavior of the Te/IGZO H‐FET. To further examine the role of the Te/IGZO heterointerface in the NDT behavior, H‐FETs with different Te/IGZO overlap lengths of 50, 100, and 150 µm were evaluated while maintaining a constant overlap width of 300 µm (Figure S8). Increasing the overlap length systematically suppresses the peak and subsequent current levels while preserving the characteristic non‐monotonic transfer behavior, indicating that the H‐FET transport characteristics are strongly dependent on the extent of the Te/IGZO heterointerface. These overlap‐dependent characteristics provide additional electrical evidence supporting the heterointerface‐mediated origin of the NDT behavior. Furthermore, the gate leakage current of the H‐FET remains substantially lower than the channel current over the relevant operating range (Figure S9), indicating that gate leakage does not make a significant contribution to the observed NDT characteristics. Figure 2i,j show the output characteristics of the Te/IGZO H‐FET measured at *V*_GS_ of −18 to 18 V in steps of 4 V, presented in the logarithmic and linear regimes, respectively. These output curves present p‐n diode‐like operation, where a rapid increase in *I*_D_ is observed as the device operates in the range of *V*_TH_n_ < *V*_GS_ < *V*_TH_p_, followed by a decrease in *I*_D_ beyond *V*_TH_p_ as the Te channel becomes depleted. Importantly, the H‐FET does not operate as a simple binary switch but instead provides a gradual and controllable increase in *I*_D_ over a wide gate‐voltage window. This analog‐like transfer characteristic enables the pull‐down current to be finely tuned, allowing the output node in the vertically stacked circuit to stabilize at intermediate voltage levels when balanced with the pull‐up CE‐Te FET. As a result, the Te/IGZO H‐FET functions as a key current‐modulating element that enables deterministic ternary‐state formation in the proposed MVL architecture. The statistical analysis of the Te/IGZO H‐FETs across over 30 devices reveals the uniformity of the proposed device (Figure S10).

### 2.3 CE‐Te FET‐Enabled Optimized MVL Operation

While the Te/IGZO H‐FET enables continuously tunable pull‐down conduction through interfacial band engineering, the realization of reliable MVL operation critically requires complementary optimization of the pull‐up transistor. A key limitation in Te‐based transistors for MVL arises from the intrinsically high off‐state current of Te channels [39, 45]. Without effective off‐current engineering, stable intermediate states may be formed through transconductance matching within the negative differential transconductance regime of the H‐FET; however, excessive leakage from the pull‐up path disrupts the pull‐down operation, leading to ambiguous ternary output levels. Therefore, precise control of the electrical characteristics of the pull‐up CE‐Te FET is essential to ensure well‐defined and robust MVL behavior. To address this challenge, we systematically engineered the Te channel to realize a high‐performance pull‐up CE‐Te FET through controlled UV‐ozone‐induced oxidation followed by Al_2_O_3_‐mediated crystallinity recovery, enabling simultaneous suppression of off‐state leakage and stabilization of the transistor characteristics. Figure 3a,b investigate the controlled surface oxidation and subsequent crystallinity recovery of the Te channel as an intentional materials engineering strategy. Time‐dependent UV‐ozone treatment was employed to induce a thin TeO_X_ layer on the Te surface, enabling precise modulation of the channel properties. As shown in Figure 3a, XPS analysis of the Te 3d core levels reveals a progressive evolution of oxidized Te^4+^ states with increasing exposure time. Quantitative peak deconvolution further shows that the Te^4+^ fraction systematically increases from 22.2% to 25.8%, 36.4%, and 61.6% with increasing UV‐ozone treatment time from 0 to 2, 4, and 6 min, respectively, confirming the progressive formation of TeO_X_. Following the Al_2_O_3_‐mediated treatment, the corresponding Te^4+^ fractions are substantially reduced to 0%, 9.3%, 22.8%, and 27.7%, respectively. This chemical‐state evolution indicates that UV‐ozone treatment progressively oxidizes the Te channel, whereas subsequent ALD Al_2_O_3_ deposition induces substantial TMA‐mediated reduction of TeO_X_ while retaining a controlled oxide composition. To mitigate excessive oxidation and to stabilize the oxidized surface, an ALD‐grown Al_2_O_3_ layer was subsequently deposited. The substantial decrease in the Te^4+^ component after Al_2_O_3_ deposition is attributed primarily to TMA‐mediated chemical reduction of TeO_X_ during the ALD process [46]. The accompanying structural evolution is separately evaluated from the XRD‐derived crystalline grain size, as discussed below.

**FIGURE 3:**
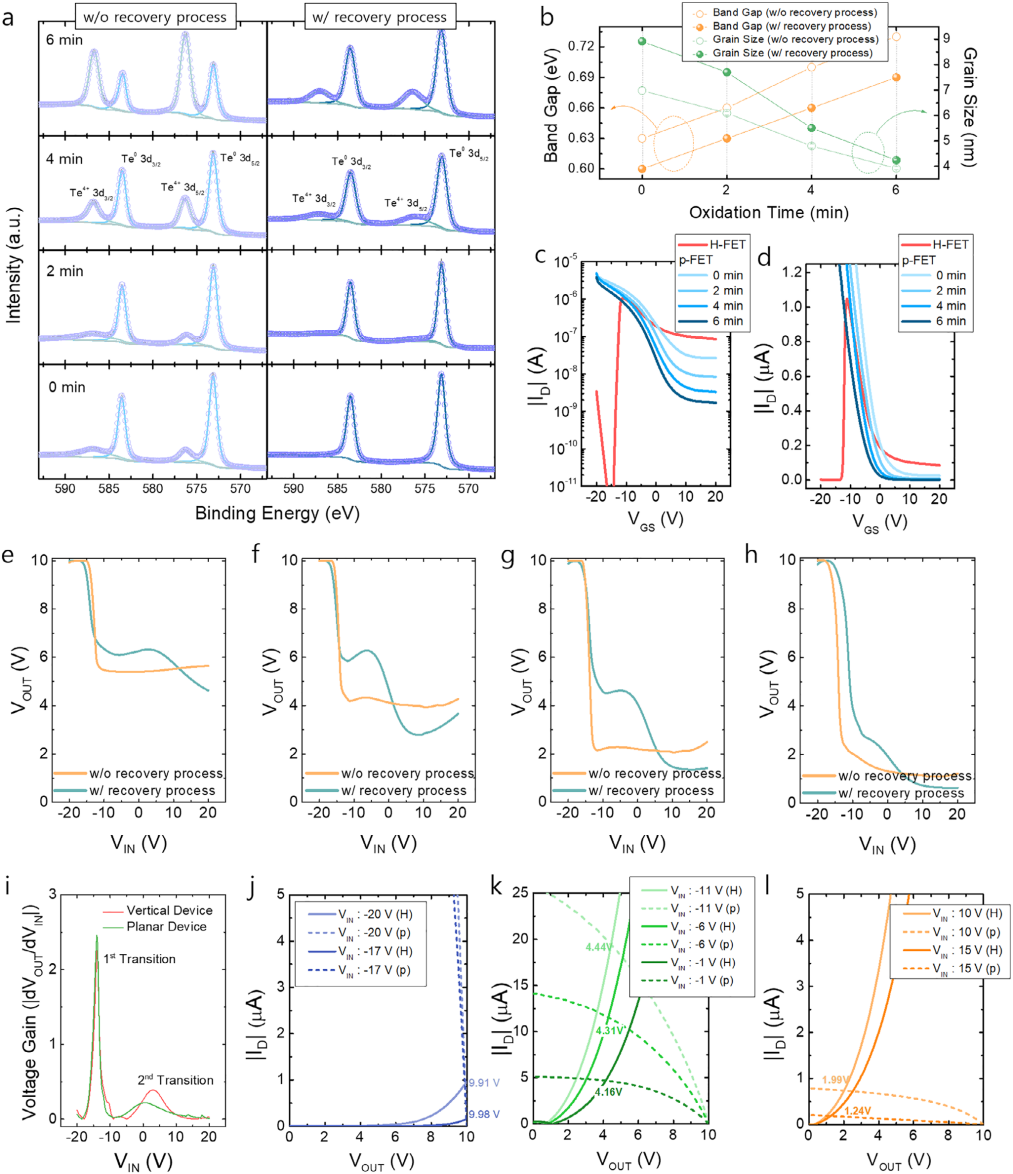
Channel engineering of the pull‐up CE‐Te FET and optimized MVL operation. (a) Te 3d XPS spectra of Te films subjected to oxidation process for different durations (0, 2, 4, and 6 min). (b) Extracted bandgap and crystalline grain size of Te channels as a function of oxidation time with and without Al_2_O_3_‐mediated crystallinity recovery treatment. (c,d) Transfer characteristics of the vertically integrated pull‐down H‐FET and pull‐up CE‐Te FET measured after complete 3D stacking, displayed in logarithmic (c) and linear (d) scales, demonstrating improved off‐state current suppression and SS of the CE‐Te FET with optimized channel engineering. (e–h) Voltage transfer characteristics of MVL circuits composed of the H‐FET and CE‐Te FET for oxidation times of 0 min (e), 2 min (f), 4 min (g), and 6 min (h) combined with crystallinity recovery. (i) Voltage gain as a function of input voltage for optimized MVL circuits in vertically integrated and planar configurations. (j–l) Current–output voltage characteristics of the pull‐down H‐FET (solid lines) and pull‐up CE‐Te FET (dashed lines) at various input voltages, illustrating current intersection points corresponding to the high (j), intermediate (k), and low (l) logic states.

The impact of controlled oxidation and subsequent Al_2_O_3_‐mediated crystallinity recovery on the electronic and structural properties of the Te channel is summarized in Figure 3b. With increasing oxidation time, the optical bandgap of Te extracted from UV–vis absorption‐based Tauc plots (Figure S11) systematically increases, reflecting the progressive incorporation of oxide species. The crystalline grain size extracted from XRD peaks (Figure S12) correspondingly decreases, indicating oxidation‐induced disruption of the crystalline Te domains. In contrast, following Al_2_O_3_ deposition, the treated Te samples exhibit slightly reduced bandgaps by approximately 0.04 eV and consistently larger XRD‐derived grain sizes than the corresponding samples without Al_2_O_3_ treatment. Together with the substantial reduction in the Te^4+^ fraction, these results indicate that TMA‐mediated deoxidation of TeO_X_ is accompanied by partial recovery of the Te‐rich crystalline domains. Importantly, the decrease in the Te^4+^ component is interpreted primarily as chemical reduction of TeO_X_, whereas the accompanying increase in XRD‐derived grain size supports the recovery of channel crystallinity. Collectively, these results establish the multi‐step process as an effective strategy for engineering the pull‐up channel through controlled modulation of the surface chemistry, band structure, and crystallinity. Progressive TeO_X_ formation during UV‐ozone treatment suppresses excessive channel conduction, whereas the subsequent Al_2_O_3_‐mediated treatment induces TMA‐mediated deoxidation accompanied by partial recovery of the crystalline Te domains. The latter is supported by the increased XRD‐derived grain size after Al_2_O_3_ deposition, while the optimized CE‐Te film maintains a smooth and continuous surface morphology (Figure 1h). These coupled chemical and structural modifications provide an optimized balance between leakage‐current suppression and sufficient channel conduction, thereby establishing the material basis for the improved electrical characteristics of the pull‐up CE‐Te FET discussed below.

Figure 3c,d present the transfer characteristics of the vertically integrated H‐FET and pull‐up p‐type CE‐Te FET after the complete stacking process, displayed in logarithmic and linear scales, respectively. The pull‐up CE‐Te FET exhibits a pronounced reduction in off‐state current and a systematic improvement in subthreshold swing (SS) with increasing oxidation time. These enhancements directly correlate with the controlled oxidation and subsequent Al_2_O_3_‐mediated crystallinity recovery identified from the chemical and structural analyses in Figure 3a,b. Progressive TeO_X_ formation effectively suppresses excessive off‐state conduction, while the subsequent Al_2_O_3_‐mediated treatment partially recovers the crystalline Te domains and provides appropriate channel conduction. These coupled modifications are reflected in the systematic evolution of the FET characteristics, including reduced off‐state current, improved subthreshold swing, and optimized transconductance matching with the pull‐down H‐FET. Consequently, the engineered CE‐Te FET enables stable current competition with the H‐FET, which is essential for reliable MVL operation. Considering the overlap‐dependent transport characteristics discussed in Figure 2, an H‐FET with a Te/IGZO overlap length of 100 µm was employed in the vertically integrated MVL circuit to provide an appropriate current level for matching with the pull‐up CE‐Te FET. The resulting transfer characteristics facilitate robust current matching between the two vertically integrated transistors, thereby ensuring stable pull‐down behavior across the operating voltage range. For comparison, the transfer curves of pull‐up Te FET subjected only to the oxidation process, together with the corresponding H‐FET characteristics, are provided in Figure S13. In the absence of crystallinity recovery treatment, excessive oxidation leads to slightly poor transconductance compatibility with the H‐FET, resulting in ineffective current balancing. The stable operation of a ternary logic inverter critically relies on the transconductance matching between the NDT regime of the H‐FET and the SS regime of the pull‐up CE‐Te FET, thereby, the proposed devices exhibit markedly improved transconductance alignment with the H‐FET, enabling well‐defined intermediate equilibrium states. Consistent transfer characteristics observed across more than 30 optimized CE‐Te FETs further demonstrate the device‐to‐device consistency of the engineered pull‐up transistor (Figure S14). This optimized matching establishes the electrical foundation for reliable and reproducible ternary logic operation in the vertically stacked MVL architecture. Furthermore, this vertical integration enables functional complementarity within a minimal lateral footprint, allowing the logic function to be implemented through vertical current competition rather than planar device duplication.

Figure 3e–h present the voltage transfer characteristics (VTCs) of the 3D vertically integrated MVL circuit composed of the pull‐down H‐FET and the pull‐up CE‐Te FET, fabricated using different surface oxidation (0‐6 min exposure) with and without the subsequent Al_2_O_3_‐mediated crystallinity recovery treatment. For each oxidation condition, devices incorporating the recovery treatment consistently exhibit superior MVL behavior compared with their corresponding untreated counterparts, highlighting the critical role of surface modulation in optimizing the pull‐up transistor performance. In the MVL circuits constructed with pristine Te and mildly oxidized Te (2 min exposure), shown in Figure 3e,f, respectively, insufficient oxidation results in elevated off‐state current in the pull‐up CE‐Te FET. Consequently, the pull‐up transistor fails to fully turn off, which interferes with the pull‐down operation of the H‐FET and degrades logic state separation. Moreover, although both the pull‐down and pull‐up currents decrease within the NDT regime of the H‐FET, their mismatched current levels prevent effective equilibrium, leading to an excessively high and poorly defined intermediate voltage state. In contrast, the MVL circuit based on CE‐Te FET with 4 min surface oxidation (Figure 3g) exhibits a well‐balanced current level comparable to that of the pull‐down H‐FET, while both currents decrease synchronously in the NDT regime. This optimized transconductance matching stabilizes the intermediate logic state and simultaneously suppresses the off‐state leakage of the pull‐up transistor, enabling robust pull‐down operation and clear ternary voltage levels. For the MVL device fabricated with extended oxidation (6 min, Figure 3h), the pull‐down operation remains effective owing to the sufficiently reduced off current of the pull‐up transistor; however, excessive oxidation significantly lowers the pull‐up current level. As a result, the current balance required for stable intermediate‐state formation is disrupted, leading to degraded MVL performance. These results collectively identify the 4 min oxidation combined with Al_2_O_3_‐mediated crystallinity recovery as the optimal channel‐engineering condition for achieving high‐performance MVL operation. Under this condition, sufficient TeO_X_ formation suppresses the off‐state leakage of the pull‐up transistor, while the subsequent Al_2_O_3_‐mediated treatment partially recovers the crystalline Te domains and maintains an appropriate pull‐up current level. This balance provides optimal transconductance matching between the pull‐up CE‐Te FET and the pull‐down H‐FET, thereby stabilizing the intermediate voltage state and enabling reliable ternary logic operation.

Using the representative VTC of the optimized MVL inverter shown in Figure 3g, the operational input voltage (*V*_IN_) ranges corresponding to the three ternary logic states were determined. The input boundaries were defined by the intersections between the VTC and the decision voltages between adjacent output‐state levels, yielding *V*_IN_ ranges of −20 to −13.95 V, −13.95 to 3.21 V, and 3.21 to 20 V for the high (+1), intermediate (0), and low (−1) logic states, respectively. The power consumption characteristics of the optimized vertically integrated MVL inverter were further evaluated based on the measured supply current (Figure S15). The static power consumption of the three stable logic states (+1, 0, and −1) was determined to be 0.5, 38.1, and 1.0 µW, respectively, corresponding to an average static power consumption of 13.2 µW. During logic‐state transitions, the maximum dynamic power consumption reached 120 µW. These results indicate that the optimized vertically integrated MVL inverter maintains low static power consumption while exhibiting only a transient increase in power during logic‐state transitions. The supply‐voltage dependence of the optimized vertically integrated MVL inverter was also examined by measuring the VTCs at *V*_DD_ values of 2.5, 5, 7.5, and 10 V (Figure S16). The characteristic multi‐step transfer behavior is retained across the investigated *V*_DD_ range, although the output voltage swing and intermediate‐state separation gradually decrease with decreasing *V*_DD_. The clearest separation of the three output states is obtained at *V*_DD_ = 10 V, indicating a trade‐off between reduced supply voltage and logic‐state separation. The thermal stability of the optimized vertically integrated MVL inverter was further assessed through temperature‐dependent VTC measurements performed at room temperature, 60°C, and 90°C (Figure S17). Although the intermediate‐state voltage exhibits a moderate decrease with increasing temperature, three well‐defined logic states are preserved throughout the investigated temperature range, demonstrating stable ternary logic functionality under practical operating conditions. To further verify the universality of the proposed MVL architecture, equivalent devices were implemented in a planar configuration. The fabrication flow of the planar MVL circuit is shown in Figure S18, together with the corresponding transfer characteristics of the pull‐down H‐FET and pull‐up CE‐Te FET in Figure S19, and the resulting VTC curves in Figure S20. The planar devices exhibit similar trends in current modulation and ternary voltage‐state formation to those of the vertically integrated architecture, while maintaining high‐performance characteristics. Figure 3i presents the voltage gain of the optimized MVL circuit as a function of *V*_IN_, demonstrating comparable performance between the vertically integrated and planar configurations. This consistency confirms that the engineered H‐FET/CE‐Te FET pair intrinsically enables MVL operation, with 3D vertical integration providing additional benefits in footprint reduction and integration density.

Figure 3j–l provide a detailed mechanistic insight into the MVL operation through *I*_D_‐output voltage (*V*_OUT_) characteristics measured at various regimes. The solid lines correspond to the pull‐down H‐FET *I*_D_s, whereas the dashed lines represent the pull‐up CE‐Te FET *I*_D_s. The intersection points between the two curves determine the steady‐state output voltage, where the pull‐up and pull‐down currents are balanced. At low input voltages (*V*_IN_ = −20 and −17 V, Figure 3j), the H‐FET remains in the off state with negligible current, while the CE‐Te FET exhibits strong conduction. As a result, the output node is biased toward the supply voltage (*V*_DD_), corresponding to the high logic state “+1”. For intermediate input voltages (*V*_IN_ = −11, −6, and −1 V, Figure 3k), the H‐FET enters its NDT regime. In this region, both the pull‐down and pull‐up currents decrease concurrently with increasing *V*_GS_, enabling their intersection to stabilize near half of the *V*_DD_. This synchronized current reduction establishes a robust intermediate equilibrium state, forming the middle logic level “0” of the MVL circuit. At high input voltages (*V*_IN_ = 10 V and 15 V, Figure 3l), the H‐FET current exceeds that of the pull‐up CE‐Te FET, driving the output voltage toward ground and realizing the pull‐down operation associated with the low logic state “−1”. Collectively, these results demonstrate that the MVL inverter operates through deterministic current competition between the H‐FET and CE‐Te FET, where the engineered NDT behavior of the pull‐down device and the optimized subthreshold characteristics of the pull‐up transistor jointly define the three stable output states. This current‐intersection‐based mechanism provides a clear physical framework for achieving reliable MVL within the 3D vertically stacked architecture.

### 2.4 Wafer‐Scale Integration of High‐Density 3D MVL Architecture

Figure 4a is a photograph of the MVL circuits fabricated at the 4‐inch wafer scale, demonstrating the large‐area uniform deposition and monolithic integration enabled by the low‐temperature, CMOS‐compatible process. The successful realization of densely distributed MVL units across the wafer highlights the scalability and practical manufacturability of the proposed vertically stacked architecture. To evaluate device‐to‐device uniformity, Figure 4b shows the VTCs of 50 randomly selected vertically integrated MVL inverters across the wafer. All measured devices exhibit well‐defined three logic states with highly consistent switching behavior, indicating robust circuit operation and good device‐to‐device reproducibility. The gate leakage characteristics of the constituent H‐FET and CE‐Te FET, together with those of the complete vertically integrated MVL inverter, are provided in Figure S9, showing low leakage currents over the operating voltage range. The corresponding statistical distributions of the *V*_OUT_s for the high (+1), intermediate (0), and low (−1) states are summarized in Figure 4c, where the narrow spread of each state confirms high uniformity and precise control of the ternary output levels. Long‐term operational stability is further assessed in Figure 4d, which displays the VTC curves of optimized MVL devices stored under air ambient conditions for up to 16 weeks. The devices maintain clear ternary characteristics without noticeable degradation in voltage levels or transfer behavior, demonstrating strong environmental stability. The temporal evolution of the *V*_OUT_ values corresponding to the three logic states is quantitatively tracked in Figure 4e on a logarithmic time scale, where all states remain stable and well separated throughout the measurement period, with highly uniform voltage gains maintained over 16 weeks (Figure S21). Collectively, these results establish the wafer‐scale scalability, high uniformity, and long‐term reliability of the vertically stacked MVL circuits, underscoring their potential for practical implementation in large‐area, high‐density multi‐valued logic systems.

**FIGURE 4:**
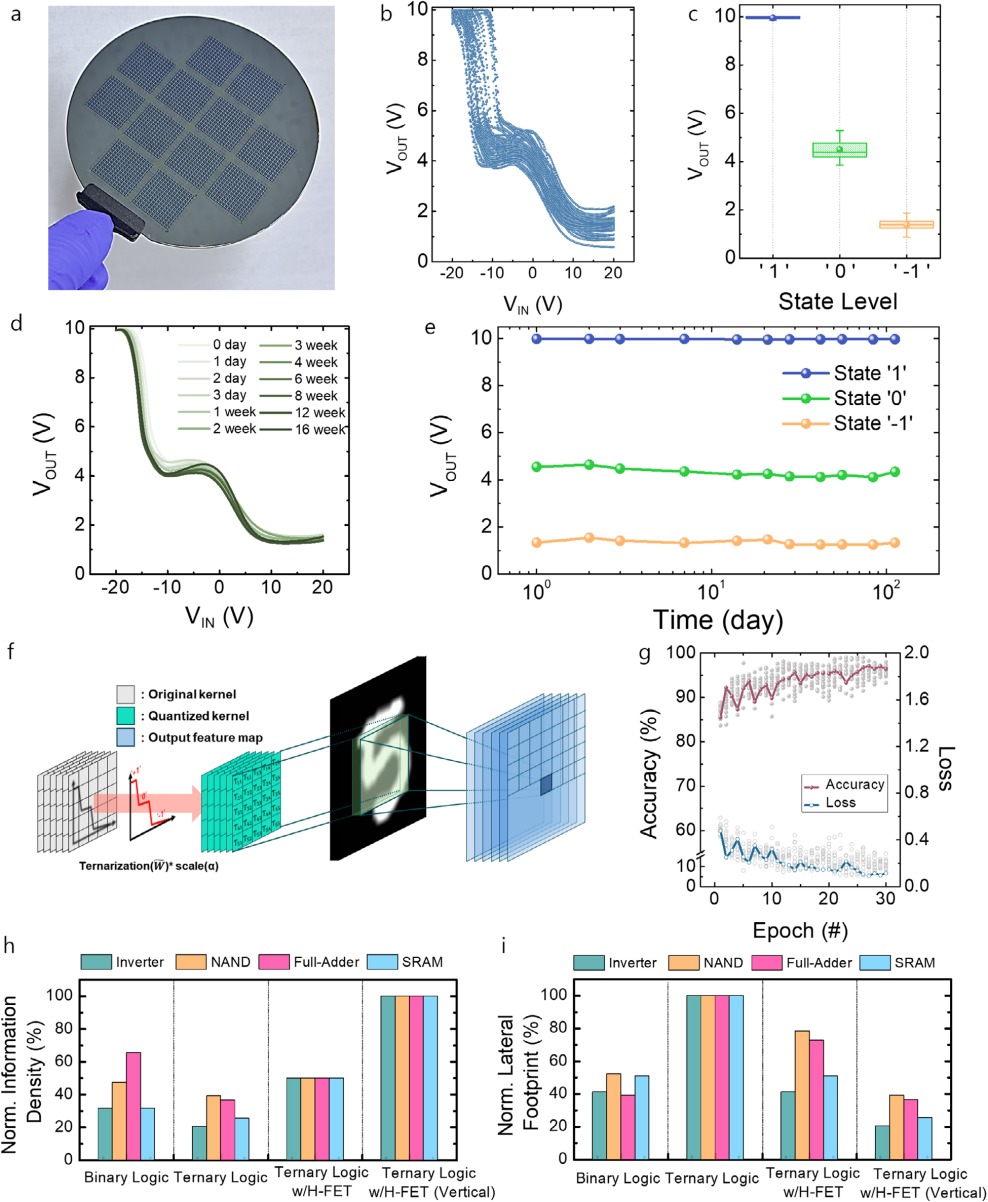
Wafer‐scale scalability, robustness, neuromorphic applicability, and density advantages of vertically stacked MVL circuits. (a) OM image of MVL circuits fabricated across a 4‐inch wafer using a low‐temperature, CMOS‐compatible monolithic process. (b) VTCs of 50 randomly selected MVL inverters across the wafer. (c) Statistical distributions of the *V*_OUT_s corresponding to the high (+1), intermediate (0), and low (−1) logic states, confirming precise and uniform ternary level formation. (d) long‐term VTC curves of optimized MVL devices measured over 16 weeks. (e) Temporal evolution of the *V*_OUT_s for the three logic states plotted on a logarithmic time scale, showing stable and well‐separated voltage levels throughout the measurement period. (f) Schematic illustration of the application of the vertically stacked ternary MVL circuits to a TWN constructed using a LeNet‐5 CNN architecture for MNIST classification. (g) Accuracy of the TWN model based on the proposed ternary voltage transfer characteristics, achieving 96.3 ± 1.16% accuracy after 30 epochs. (h) Histogram comparison of normalized information density for binary and ternary inverters, NAND gates, full adders, and SRAM cells with and without H‐FET‐assisted 3D integration. i) Corresponding histogram of normalized lateral footprint, highlighting substantial footprint reduction enabled by the proposed vertically stacked ternary architecture.

Figure 4f illustrates the conceptual integration of the vertically stacked ternary MVL circuits into a ternary weight network (TWN) for neuromorphic computing applications. To evaluate the computational potential of the proposed ternary circuits, a convolutional neural network (CNN) for handwritten digit recognition was constructed based on the LeNet‐5 architecture, a widely adopted framework for image classification tasks [11, 47]. In this implementation, network weights were constrained to three discrete values {−1, 0, +1} using the TWN approach, where each ternary weight is further scaled by a factor α to preserve representational fidelity while enabling substantial model compression of up to sixteenfold [48]. The experimentally measured ternary VTCs of the proposed MVL inverter were used to define the three quantized weight states of the TWN, providing an experimentally calibrated software framework for evaluating the applicability of the proposed architecture to ternary neuromorphic computing. The CNN model employed three convolutional layers, two pooling layers, and two fully connected layers (see Figure S22, for detailed matrix dimensions). During training, ternarization was selectively applied to the convolutional and fully connected layers, where weight updates play a dominant role in network inference and learning. The TWN achieved an average MNIST classification accuracy of 96.3 ± 1.16% over multiple independent training runs after 30 epochs (Figure 4g), demonstrating good reproducibility of the experimentally calibrated ternary neural‐network model. These results demonstrate that the ternary logic behavior realized by the vertically stacked MVL circuits can effectively support multi‐level weight representation in deep learning frameworks without substantial degradation in classification performance. The corresponding confusion matrix, presented in Figure S23, confirms high‐precision recognition across all digit classes, further validating the robustness of the ternary‐weight‐based inference. This demonstration highlights the potential applicability of the proposed vertically integrated ternary circuit to future hardware neuromorphic systems by bridging experimentally demonstrated device‐level multi‐state operation with software‐level neural‐network evaluation, providing a promising pathway toward energy‐efficient and memory‐efficient deep neural network acceleration.

Figure 4h,i quantitatively evaluate the area efficiency and logic density advantages enabled by the proposed H‐FET‐based 3D integration scheme. Histograms compare normalized information capacity and normalized lateral footprint across binary and ternary inverters, NAND gates, full adders, and SRAM cells, considering both the presence of the H‐FET and the implementation of 3D vertical stacking. The normalized lateral footprint was estimated from the equivalent lateral active‐channel footprint under an identical unit‐channel‐area assumption (300 µm × 400 µm per transistor), where vertically stacked transistors were counted as two physical devices but one equivalent lateral footprint. The corresponding physical transistor counts and equivalent lateral footprints for each logic implementation are summarized in Table S2, including binary, ternary, planar H‐FET‐based ternary, and vertically integrated H‐FET‐based ternary architectures. The normalized information capacity further accounts for the information content per logic symbol, corresponding to log_2_(2) = 1 bit for binary logic and log_2_(3) = 1.585 bits for ternary logic. Detailed calculation procedures and layout assumptions are provided in the Supporting Information. The proposed 3D vertically stacked MVL logic achieves a sixfold normalized logic density compared with conventional planar ternary logic without H‐FET integration. These gains arise from the simultaneous reduction in transistor count enabled by MVL operation and the footprint compression achieved through vertical integration. Substantial improvements in both footprint occupation and information capacity are consistently obtained for more complex logic blocks, including NAND gates, full adders, and SRAM cells. This scalability demonstrates that the proposed architecture provides a generalizable pathway for realizing high‐density computing systems rather than a device‐specific optimization. Taken together, these results establish that the vertically stacked MVL platform achieves an exceptional combination of ultracompact footprint and high logic density, highlighting its strong potential for overcoming conventional planar scaling limitations and enabling next‐generation high‐density integrated circuits.

## 3 Conclusions

In this work, we have demonstrated a monolithic 3D MVL platform based on vertically stacked heterojunction and p‐type transistors. By integrating a pull‐down Te/IGZO H‐FET with a pull‐up p‐type CE‐Te FET in a vertical architecture, we translated interfacial band alignment and engineered current modulation directly into circuit‐level ternary logic functionality. The carrier‐selective type‐I band alignment of the H‐FET enabled gate‐tunable electron‐dominated transport and negative differential transconductance behavior by confining carriers within the heterointerface region, which is particularly advantageous for stabilizing intermediate equilibrium states essential for MVL operation, while controlled oxidation and crystallinity recovery of the CE‐Te channel effectively suppressed off‐state leakage and optimized transconductance matching. This synergistic device engineering resulted in robust and deterministic ternary circuit operation, with stable intermediate equilibrium states governed by current intersection between the vertically integrated transistors. Beyond individual device performance, we achieved wafer‐scale implementation using a low‐temperature, CMOS‐compatible process, demonstrating high uniformity, long‐term operational stability, and scalable manufacturability. System‐level evaluations further revealed substantial improvements in area efficiency and logic density enabled by H‐FET‐assisted 3D integration, extending from basic inverters to complex logic blocks and memory elements. Moreover, the compatibility of the ternary VTC with TWN enabled practical neuromorphic computing demonstrations, achieving high‐accuracy MNIST classification and highlighting the potential of multi‐valued hardware for memory‐efficient and energy‐efficient deep learning acceleration. Taken together, this vertically stacked MVL architecture establishes a scalable pathway toward high‐density integrated circuits that transcend conventional binary planar scaling. By unifying interfacial band engineering, channel modulation, and 3D integration, our approach offers a versatile platform for next‐generation logic systems and multi‐valued neuromorphic computing technologies.

## 4 Experimental Section

### 4.1 Device Fabrication

Vertically stacked MVL circuits were fabricated on heavily n‐doped Si substrates with a thermally grown SiO_2_ layer. All processes were conducted at temperatures below 200°C to ensure CMOS compatibility. For the pull‐down H‐FET, n‐type IGZO (20 nm thick, width/length (W/L) of 300 µm/200 µm) and p‐type Te (10 nm thick, W/L of 300 µm/300 µm) thin films were sequentially deposited by sputtering with a Te/IGZO overlap region of 300 µm × 100 µm to form the heterointerface and patterned by photolithography. ITO source and drain electrodes (30 nm thick) were subsequently sputter‐deposited, followed by photolithographic patterning and lift‐off. The stacked heterojunction structure was air‐annealed at 200°C step for 1 h. A 50‐nm‐thick Al_2_O_3_ layer was then deposited by ALD as the first gate dielectric using trimethylaluminum (TMA) and H_2_O serving as the Al and O precursors, respectively. A common gate electrode was formed by sputtering 30‐nm‐thick ITO, followed by deposition of a second 50‐nm‐thick ALD Al_2_O_3_ layer by ALD to electrically isolate the vertically integrated transistors. Gate via openings (50 nm depth) and H‐FET source/drain via openings (100 nm depth) were defined by wet etching process to enable vertical interconnection. For the pull‐up CE‐Te FET, 8‐nm‐thick Te channel layer was sputter‐deposited onto the upper dielectric surface. Controlled surface oxidation was induced by UV‐ozone treatment for varying durations (0–6 min) to modulate surface chemistry and electronic properties. After the oxidation process, the pull‐up source electrode, common drain electrode, and gate pads were deposited using sputtered ITO and patterned by photolithography. The stacked devices were annealed at 200°C to enhance electrical contact and interfacial stability. Subsequently, Al_2_O_3_ layer was deposited by ALD, which facilitated crystallinity recovery and partial reduction of the oxidized Te layer through chemical interaction with the TMA precursor, followed by a low‐temperature annealing step at 150°C for 1 h and pad opening etching to complete the device fabrication. Planar MVL devices used for comparison were fabricated following identical material deposition and engineering processes without vertical stacking.

### 4.2 Neural Network Training and Ternary Weight Quantization

The neuromorphic performance of the vertically stacked ternary logic circuits was evaluated using a TWN implemented with a CNN based on the LeNet‐5 architecture. The standard MNIST handwritten digit dataset was employed for training and validation. The batch size was set to 128, and the network was trained for 30 epochs. The input grayscale images of 28 × 28 pixels were first zero‐padded to 32 × 32 pixels. The first convolutional layer applied six 5 × 5 kernels, followed by a 2 × 2 average pooling operation, resulting in feature maps of size (6, 14, 14). A second convolutional layer with sixteen 5 × 5 kernels was subsequently applied, followed by another 2 × 2 average pooling layer to yield feature maps of size (16, 5, 5). A third convolutional layer consisting of 120 kernels of size 5 × 5 was then applied, producing feature maps of size (120, 1, 1). The resulting feature maps were subsequently flattened into a 120‐dimensional feature vector and passed through fully connected layers containing 84 and 10 neurons, respectively, for classification into ten digit categories. To emulate the electrical characteristics of the proposed MVL circuit, network weights were constrained to three discrete states {+1, 0, −1} through a ternary quantization scheme. The floating‐point weight *W_i_* was mapped to a ternary value ${\tilde{W}}_{i}$ according to predefined thresholds:

$$+1\;if\;W_{i}\;<\;W_{min}+2\Delta \;$$

$$\tilde{W}=f\;\left(W_{i}|\Delta \right)=0\;if\;W_{min}+2\Delta \;\leq W_{i}<\;W_{max}-\;3\Delta \;$$

$$-1\;if\;W_{i}\leq W_{max}\;-\;3\Delta \;$$

$$\left(\Delta =\left(W_{max}-W_{min}\right)/8\right)$$

To preserve magnitude information and minimize the Euclidean distance between the full‐precision and ternarized weights, a scaling factor α was applied for each filter:

$$\alpha =\left\{\begin{array}{c} \frac{W\tilde{\cdot W}}{\tilde{W}\cdot \tilde{W}},\;\;if\;\tilde{W}\cdot \tilde{W}\;\neq \;0\\ 0,\;\;if\tilde{\;W}\cdot \tilde{W}=0 \end{array}\right.$$

The ternarized and scaled weights were utilized during both forward and backward propagation, while gradient updates were accumulated on the full‐precision floating‐point weights to ensure stable convergence. This training framework enabled direct correspondence between the three discrete logic states of the hardware ternary inverter and the quantized weight values in the TWN, allowing evaluation of the proposed multi‐valued logic circuits in neuromorphic computing tasks.

### 4.3 Characterizations and Measurements

Structural properties of the Te, IGZO, and heterojunction films were investigated by XRD using a Dmax2500/PC diffractometer (Rigaku) with Cu Kα radiation (λ = 1.54059 Å). Diffraction patterns were recorded to analyze crystallographic phases and peak broadening, from which grain sizes were estimated using the Debye–Scherrer equation. OM images of wafer‐scale devices and fabrication process steps were captured using an optical microscope (BX53M, Olympus, Japan). Optical absorption measurements were carried out using a UV–vis spectrophotometer (V‐670, JASCO). The absorption spectrum was used to extract optical bandgaps of the Te and oxidized Te channels through Tauc plot analysis. XPS analysis was performed using a Nexsa system (Thermo Fisher Scientific, USA) with an Al Kα X‐ray source (photon energy of 1486.6 eV). Core‐level spectra were used to analyze band alignment and interfacial chemistry. The valence band spectra were collected to determine the valence band maxima of Te and IGZO. All spectra were referenced to the C 1s level. Raman spectra were acquired using a DXR2xi Raman system (Thermo Fisher Scientific) with a 532 nm excitation laser and a laser power maintained at 8 mW. Spectra were acquired at a frequency of 10 Hz with 400 accumulated scans to improve signal‐to‐noise ratio. Cross‐sectional microstructural analysis was performed using a FE‐TEM (JEM‐F200 (TFEG), JEOL, Japan) with a spatial resolution of 0.10 nm. Elemental distributions were mapped using an integrated EDS with a dual silicon drift detector (SDD) with an effective solid angle of 1.7 sr. Focused ion beam (FIB) prepared TEM specimens (Crossbeam 540, ZEISS) were mounted on Cu grids. Surface morphology and thickness profiles of the deposited films were characterized using an atomic force microscope (NX‐10, Park Systems, Korea, located at the 3D Convergence Center of Inha University) operated in non‐contact mode. Electrical measurements were conducted using a semiconductor parameter analyzer (Agilent 4156C), recording the transfer and output characteristics and the VTCs of the MVL circuits under ambient air conditions at room temperature. Long‐term stability measurements were performed by periodically monitoring device performance over extended storage in air ambient.

## Author Contributions

C.‐H.K. and I.‐J.P. conceived the idea and designed the overall device architecture and experiments. C.‐H.K. fabricated the vertically stacked devices and performed electrical measurements. M.‐S.K. and T.I.K. conducted the materials characterizations and analyses. C.‐H.K. and J. B. Rhim developed and trained the neural network models and performed neuromorphic computing simulations. C.‐H.K., T.I.K., and I.‐J.P. analyzed the data. T.I.K., H.‐I.K., and I.‐J.P. wrote the manuscript. H.‐I.K. and I.‐J.P. supervised the project and provided critical feedback on the research direction and manuscript preparation. All authors discussed the results and approved the final version of the manuscript.

## Conflicts of Interest

The authors declare no conflicts of interest.

## Supporting information




**Supporting File**: advs77437‐sup‐0001‐SuppMat.docx.

## Data Availability

The data that support the findings of this study are available from the corresponding authors upon reasonable request.
