# Supplementary material for: Wafer‐Scale 3D Integration for High‐Density Multi‐Valued Neuromorphic Logic
Source: Adv Sci (Weinh). 2026 Aug 25;13(63):e77437. doi: 10.1002/advs.77437 (PMC13505468; doi:10.1002/advs.77437)
Supplement: Supplementary file 1 [file ADVS-13-e77437-s001.docx]

Supporting Information

**Wafer-Scale 3D Integration for High-Density Multi-Valued Neuromorphic Logic**

Chang-Hyeon Kim, Min Seok Kim, Joong Bum Rhim, Tae In Kim^*^, Hyuck-In Kwon^*^, and Ick-Joon Park^*^

C.-H. Kim, H.-I. Kwon

School of Electrical and Electronics Engineering, Chung-Ang University, Seoul, 06974, Korea

E-mail: hyuckin@cau.ac.kr

M. S. Kim

Department of Electrical and Electronic Engineering, Joongbu University, Goyang, 10279, Korea

J. B. Rhim

Department of Electrical Engineering and Computer Science, Massachusetts Institute of Technology, Cambridge, MA 02139, USA

T. I. Kim

School of Electrical and Electronic Engineering, Program in Semiconductor Convergence, Inha University, Incheon 22212, Korea

E-mail: taein.kim@inha.ac.kr

I.-J. Park

Department of Electronics and Information Engineering, Hansung University, Seoul, 02876, Korea

E-mail: ickjoon.park@hansung.ac.kr

Keywords: 3D monolithic integration, multi-valued logic, heterojunction FET, tellurium transistor, neuromorphic logic, high-density ternary circuit

**Figure S1. Optical microscopy image of the fabricated vertically integrated MVL inverter.** Optical microscopy image of the proposed MVL inverter showing the device layout, channel dimensions, Te/IGZO overlap region, via geometry, and overall footprint.

**Figure S2. AFM height profiles of the vertically stacked channel layers.** a) AFM images and corresponding height profiles, confirming thicknesses of approximately 20 nm for the pull-down IGZO layer, b) 10 nm for the pull-down Te layer, and c) 8 nm for the pull-up Te layer, respectively.

**Figure S3. Detailed Raman peak positions of Te and Te/IGZO heterojunction.** a) Raman spectra highlighting the A_1_ and E_2_ vibrational modes of pristine Te and b) the Te/IGZO heterojunction, with extracted peak positions showing slight blueshifts upon heterojunction formation while preserving the characteristic spectral features of crystalline Te.

**Figure S4. XPS core-level and valence band spectra of bulk Te.** a) Te 3d core-level spectra and b) corresponding valence band spectra of the bulk Te reference sample used to determine the binding energy separation between the Te core level and the valence band maximum for band offset extraction.

**Figure S5. XPS core-level and valence band spectra of bulk IGZO.** a) In 3d, b) Ga 2p, and c) Zn 2p core-level spectra together with d) the valence band spectra of the bulk IGZO reference sample, providing the energy separations required for quantitative band alignment analysis.

**Figure S6. Optical bandgap extraction of Te and IGZO films.** a) UV-Vis absorption spectra based Tauc plots for Te and b) IGZO thin films, used to determine their optical bandgaps for conduction band offset calculation.

**Figure S7. Electrical characteristics of individual Te and IGZO FETs.** a) Transfer curves of the standalone p-type Te FET and b) n-type IGZO FET, demonstrating their respective switching behaviors prior to heterojunction integration.

**Figure S8. Effect of Te/IGZO overlap length on the transfer characteristics of the H-FET.** Transfer characteristics of H-FETs with Te/IGZO overlap lengths of 50, 100, and 150 μm at a fixed overlap width of 300 μm (|V_DS_| = 1 V).

**Figure S9. Gate leakage characteristics of the H-FET, CE-Te FET, and vertically integrated MVL inverter.** The low leakage currents over the operating voltage range confirm the electrical integrity of the devices and exclude significant gate-leakage contributions to the observed electrical characteristics.

**Figure S10. Statistical analysis of Te/IGZO H-FET performance.** a) Transfer curves measured from over 30 Te/IGZO heterojunction FET devices and b) corresponding distribution of key electrical parameters such as peak current, c) peak voltage, and d) transconductance, confirming the uniformity and reproducibility of the proposed device architecture.

**Figure S11. Tauc plots of oxidized Te channels.** a) Tauc plots of Te films treated with different UV-ozone durations for non-recrystallized and b) recrystallized samples, used to determine oxidation-dependent bandgap evolution.

**Figure S12. XRD patterns of Te channels under oxidation and recrystallization.** a) XRD patterns of Te films subjected to various oxidation treatment times without and b) with recrystallization, showing oxidation-induced peak broadening and recrystallization-assisted recovery of crystallinity.

**Figure S13. Transfer characteristics of H-FETs and oxidation-only pull-up Te FETs.** Transfer curves of pull-up Te FETs processed with oxidation without surface recrystallization together with the pull-down H-FET characteristics, highlighting degraded current matching compared with recrystallized devices.

**Figure S14. Device-to-device uniformity of CE-Te FETs.** Transfer characteristics of more than 30 CE-Te FETs, demonstrating good device-to-device uniformity and reproducibility.

**Figure S15. Power consumption of the fabricated vertically integrated MVL inverter.** Power consumption of the proposed ternary inverter as a function of the input voltage.

**Figure S16. Supply-voltage-dependent characteristics of the vertically integrated MVL inverter.** Voltage transfer characteristics of the optimized vertically integrated MVL inverter measured at V_DD_ values of 2.5, 5, 7.5, and 10 V.

**Figure S17. Temperature-dependent voltage transfer characteristics of the fabricated vertically integrated MVL inverter.** Temperature-dependent voltage transfer characteristics of the proposed MVL inverter measured at RT, 60 °C, and 90 °C under a supply voltage of 10 V.

**Figure S18. Fabrication flow of planar MVL architecture.** Optical microscopy images illustrating the process sequence for planar integration of the pull-down H-FET and pull-up CE-Te FET.

**Figure S19. Electrical characteristics of planar devices.** Transfer characteristics of the pull-down H-FET and pull-up CE-Te FET implemented in the planar configuration.

**Figure S20. Voltage transfer characteristics of planar MVL inverters.** VTC curves of planar MVL circuits composed of the H-FET and CE-Te FET, demonstrating similar ternary behavior and performance trends to the vertically integrated architecture.

**Figure S21. Long-term voltage gain stability of MVL circuits.** Evolution of voltage gain values for the three logic states over 16 weeks under ambient storage conditions, confirming highly stable amplification behavior.

**Figure S22. Layer configurations of the LeNet-5-based ternary weight network.** Matrix dimensions and structural details of each convolutional, pooling, and fully connected layer employed in the TWN model used for MNIST classification.

**Figure S23. Confusion matrix for MNIST classification using the TWN model.** 3D Comparison between true labels and predicted labels for handwritten digit recognition, demonstrating high classification accuracy across all digit classes.

**Table S1.** **Comparison of device, circuit, and integration features of the proposed vertically integrated H-FET-based MVL platform with representative previous works.**

| **Device architecture** | **Intrinsic MVL operation** | **Monolithic vertical integration** | **Wafer-scale fabrication** | **Low-temperature processing (≤200°C)** | **Quantified logic-density benefit** | **Ref.** |
| --- | --- | --- | --- | --- | --- | --- |
| BP/ReS_2_/h-BN NDR FET | O | × | × | - | - | [S1] |
| BP/ReS_2_ NDR FET | O | × | × | - | - | [S2] |
| BP/MoS_2_ H-FET | O | × | × | - | - | [S3] |
| DNTT/PTCDI-C13 organic H-FET | O | × | × | O | - | [S4] |
| 3D organic H-FET /FG-FET | O | O | × | O | - | [S5] |
| MoTe_2_/SnS_2_ vdW H-FET | O | × | × | O | - | [S6] |
| MoS_2_/WSe_2_ H-FET | O | × | × | O | - | [S7] |
| Te/IGZO H-FET | O | × | × | O | - | [S8] |
| TeO_X_/IGTO  H-FET | O | × | O | × | - | [S9] |
| TeSeO/IGZO H-FET | O | × | × | × | - | [S10] |
| SOI/MoS_2_ CFET | × | O | O | × | - | [S11] |
| MoS_2_/CrOCl CFET | × | O | × | - | O | [S12] |
| Te/IGZO  H-FET-based 3D MVL | **O** | **O** | **O** | **O** | **O** | **This work** |

**O : experimentally demonstrated, ×: not demonstrated, -: not explicitly reported.**

**Table S2.** **Comparison of physical transistor count and equivalent lateral footprint for representative binary and ternary logic architectures.**

| **Logic Block** | **Metric** | **Binary Logic** | **Ternary Logic** | **Ternary Logic w/H-FET** | **Ternary Logic w/H-FET (Vertical)** |
| --- | --- | --- | --- | --- | --- |
| **Inverter** | Physical transistor count | 2 | 6 | 2 | 2 |
|  | Equivalent lateral footprint, (*A*_unit_) | 2 | 6 | 2 | 1 |
| **NAND** | Physical transistor count | 4 | 8 | 6 | 6 |
|  | Equivalent lateral footprint, (*A*_unit_) | 4 | 8 | 6 | 3 |
| **Full Adder** | Physical transistor count | 28 | 74 | 58 | 58 |
|  | Equivalent lateral footprint, (*A*_unit_) | 28 | 74 | 58 | 29 |
| **SRAM** | Physical transistor count | 6 | 16 | 6 | 6 |
|  | Equivalent lateral footprint, (*A*_unit_) | 6 | 16 | 6 | 3 |

All comparisons were performed using an identical unit active-channel footprint of 300 μm × 400 μm per transistor, corresponding to the fabricated device shown in Figure S1. The physical transistor count represents the total number of transistors required to implement each logic function, whereas the equivalent lateral footprint represents the number of unit active-channel areas occupied in the lateral plane. For the proposed vertically integrated architecture, vertically overlapping transistors were counted individually in the physical transistor count but were considered to occupy a single equivalent lateral footprint. The normalized lateral footprint was obtained by normalizing the equivalent lateral footprint to that of the corresponding conventional planar ternary logic implementation. Contact pads, interconnects, alignment margins, and peripheral circuitry were excluded from this active-channel-footprint-based comparison.

**References**

S1. Seo, S. *et al.* A Van Der Waals Reconfigurable Multi‐Valued Logic Device and Circuit Based on Tunable Negative‐Differential‐Resistance Phenomenon. *Adv. Mater.* **34,** 2202799 (2022). <https://doi.org/10.1002/adma.202202799>

S2. Shim, J. *et al.* Phosphorene/rhenium disulfide heterojunction-based negative differential resistance device for multi-valued logic. *Nat. Commun.* **7,** 13413 (2016). <https://doi.org/10.1038/ncomms13413>

S3. Huang, M. *et al.* Multifunctional high-performance van der Waals heterostructures. *Nat. Nanotechnol.* **12**, 1148–1154 (2017). [https://doi.org/10.1038/nnano.2017.208](https://doi.org/10.1038/nnano.2017.208 )

S4. Yoo, H., On, S., Lee, S. B., Cho, K. & Kim, J. Negative Transconductance Heterojunction Organic Transistors and their Application to Full‐Swing Ternary Circuits. *Adv. Mater.* **31**, 1808265 (2019). <https://doi.org/10.1002/adma.201808265>

S5. Choi, J. *et al.* Vertically stacked, low-voltage organic ternary logic circuits including nonvolatile floating-gate memory transistors. *Nat. Commun.* **13**, 2305 (2022). <https://doi.org/10.1038/s41467-022-29756-w>

S6. C. Chen, S. Yang, C. Lin, M. Lee, M. Tsai, F. Yang, Y. Chang, M. Li, K. Lee, K. Ueno, Y. Shi, C. Lien, W. Wu, P. Chiu, W. Li, S. Lo, Y. Lin, Reversible Charge‐Polarity Control for Multioperation‐Mode Transistors Based on van der Waals Heterostructures. *Adv. Sci.* ***9****,* 2106016 (2022). <https://doi.org/10.1002/advs.202106016>

S7. Han, K. H. *et al.* Charge transfer mechanism for realization of double negative differential transconductance. *npj 2D Mater. Appl.* **8**, 15 (2024). <https://doi.org/10.1038/s41699-024-00454-z>

S8. Lee, D. H. *et al.* A Mixture of Negative-, Zero-, and Positive-Differential Transconductance Switching from Tellurium/Indium Gallium Zinc Oxide Heterostructures. *ACS Appl. Mater. Interfaces* **16**, 20705–20714 (2024). <https://doi.org/10.1021/acsami.3c19471>

S9. Jung, C. *et al.* Scalable Ternary Logic Device Based on TeOx/IGTO Heterojunction Transistor. *Adv. Funct. Mater.* **35**, 2501834 (2025). <https://doi.org/10.1002/adfm.202501834>

S10. Jin, M., Shin, J. C., Lee, J., Hwang, S. & Kim, Y. S. All-Inorganic Metal Oxide-Based p-n Heterojunction Ternary-State Transistors. *ACS Nano* **19,** 22044–22053 (2025). <https://doi.org/10.1021/acsnano.5c01840>

S11. Tong, L. *et al.* Heterogeneous complementary field-effect transistors based on silicon and molybdenum disulfide. *Nat. Electron.* **6**, 37–44 (2023). <https://doi.org/10.1038/s41928-022-00881-0>

S12. Guo, Y. *et al.* Van der Waals polarity-engineered 3D integration of 2D complementary logic. *Nature* **630**, 346–352 (2024). <https://doi.org/10.1038/s41586-024-07438-5>
